# Creatine Supplementation Upregulates mTORC1 Signaling and Markers of Synaptic Plasticity in the Dentate Gyrus While Ameliorating LPS-Induced Cognitive Impairment in Female Rats

**DOI:** 10.3390/nu13082758

**Published:** 2021-08-11

**Authors:** Xuansong Mao, Taylor J. Kelty, Nathan R. Kerr, Thomas E. Childs, Michael D. Roberts, Frank W. Booth

**Affiliations:** 1 Department of Biomedical Sciences, University of Missouri, Columbia, MO 65211, USA; xmwhd@UMsystem.edu (X.M.); taylor.kelty@mail.missouri.edu (T.J.K.); nrkfkd@mail.missouri.edu (N.R.K.); childst@missouri.edu (T.E.C.); 2 School of Kinesiology, Auburn University, Auburn, AL 36849, USA; mdr0024@auburn.edu; 3 Department of Nutrition and Exercise Physiology, University of Missouri, Columbia, MO 65211, USA; 4 Department of Medical Pharmacology and Physiology, University of Missouri, Columbia, MO 65211, USA; 5 Dalton Cardiovascular Research Center, University of Missouri, Columbia, MO 65211, USA

**Keywords:** creatine, mild cognitive impairment, mTORC1, dentate gyrus

## Abstract

Mild cognitive impairment (MCI) designates the boundary area between cognitive function in natural aging and dementia, and this is viewed as a therapeutic window to prevent the occurrence of dementia. The current study investigated the neurocognitive effects of oral creatine (Cr) supplementation in young female Wistar rats that received intracerebroventricular injections of lipopolysaccharide (LPS) to mimic MCI. Neuromolecular changes within the dentate gyrus were analyzed following behavioral testing. We also investigated both neurocognitive and neuromolecular changes following Cr supplementation in the absence of LPS in young female Wistar rats to further investigate mechanisms. Interestingly, based on trial 2 of Barnes maze test, Cr supplementation ameliorated spatial learning and memory deficit induced by LPS, shown by decreased latency time and errors to reach the escape box (*p* < 0.0001, *n* = 12). Cr supplementation also attenuated recognition memory deficit induced by LPS, shown by increased amount of time taken to explore the new object (*p* = 0.002, *n* = 12) during novel object recognition testing. Within the dentate gyrus, Cr supplementation in LPS injected rats upregulated mTORC1 signaling (*p* = 0.026 for mTOR phosphorylation, *p* = 0.002 for p70S6K phosphorylation, *n* = 8) as well as the synapsin (*p* = 0.008) and PSD-95 synaptic proteins (*p* = 0.015), in comparisons to LPS injected rats. However, Cr supplementation failed to further enhance spatial memory and recognition memory in the absence of LPS. In conclusion, Cr ameliorates LPS-induced cognitive impairment in a rodent MCI model. Mechanistically, these phenotypic effects may, in part, be mitigated via an upregulation of mTORC1 signaling, and an enhancement in synaptogenesis in the dentate gyrus. While preliminary, these findings may inform future research investigating neurocognitive effects of Cr for MCI patients.

## 1. Introduction

Dementia is a comprehensive descriptor for various combinations of symptoms, which include deficits in memory, problem solving, thinking skills, or language [1]. The most prevalent dementia type is Alzheimer’s Disease (AD). AD has been categorized into three developmental phases that are preclinical, mild cognitive impairment (MCI) AD, and dementia AD [2]. Hence, MCI transcends between the preclinical and dementia AD stages. Although results from the Rush Memory and Aging Projects showed 42% of the MCI patients developed dementia after a median of ~3 yrs with 38% of patients reverting back to normal at the same time [3], studies have shown that the AD pathology is irreversible in nature once it occurs [4,5]. This implies the clinical significance of using MCI as an early therapeutic window for populations who are vulnerable to AD. However, therapeutic interventions for mitigating the progression of MCI are currently lacking.

Creatine (Cr) is a naturally occurring compound that is synthesized from the amino acids glycine and arginine, primarily in liver and kidneys. When Cr is stored, it is converted to high energy form of phosphocreatine (PCr), which provides immediate energy supply by donating its phosphate to regenerate ATP through the creatine kinase (CK) reaction, as energy demand increases [6]. Although Cr supplementation has been widely used as an ergogenic aid for professional and recreational athletes for decades [6]; there has been recent interest in examining its efficacy in enhancing cognition [7,8]. In a double-blinded designed human study, Rae et al. [9] found that six-weeks of Cr supplementation significantly improved working memory and intelligence score. McMorris and colleagues [10] further revealed a significant effect of Cr on enhancing cognition in elderly participants after only one week of Cr supplementation. Other studies have assessed the cognitive-enhancing properties of Cr under stressed conditions. For example, in 24 h and 36 h sleep-deprived individuals, Cr supplementation significantly augmented cognitive performance compared with placebo-control [11,12]. In addition, 7 days of Cr supplementation has been shown to restore the acute hypoxia-induced decrements in cognitive performance in healthy young adults [13]. 

While promising, the neuro-molecular mechanisms associated with the cognitive benefits underlying Cr supplementation are not well established. Mammalian target of rapamycin complex 1 (mTORC1) is a protein complex that is composed of mTOR, Raptor, mLST8, Deptor and PRAS40, and mTORC1 functions to sense and integrate nutritional and environmental cues, such as amino acids, growth factors, stress level, and energy status, to regulate many essential anabolic processes. Upon activation, mTORC1 complex promotes protein synthesis mainly through phosphorylating downstream 70-kDa ribosomal protein S6 kinase (p70S6K) and eukaryotic initiation factor 4E (eIF4E)-binding protein (4EBP) [14]. Studies have implied that mTORC1 signaling may be a mechanism involved in mediating the cognitive enhancement of Cr supplementation. For example, it has been shown that the inhibition of mTORC1 signaling via rapamycin in hippocampal preparations reduced long-term potentiation (LTP) induced by high-frequency stimulation and BDNF [15]. It has also been shown that learning tasks induce a rapid increase in the phosphorylation status of mTOR (Ser 2448) and its specific substrate p70S6K in the hippocampus, while bilateral infusion of rapamycin into CA1 region of the hippocampus diminishes the mTORC1 pathway and memory formation [16], suggesting a key role of mTORC1 signaling in mediating memory formation and learning. Others have reported Cr supplementation in mice increases hippocampal mTORC1 signaling [17,18]. Collectively, these findings suggest mTORC1 signaling could be a candidate pathway that underlies the cognitive benefits of Cr. 

Herein, we utilized an LPS-induced rodent MCI model, which exhibits impaired spatial and recognition memory, published previously by our laboratory [19], to examine potential cognitive effects of Cr supplementation. Furthermore, we examined neuro-molecular mechanisms in the dentate gyrus resulted from and associated with Cr supplementation, this being a sub-region of hippocampus known to play a fundamental role in hippocampus-dependent learning and memory [20,21]. In the current study, we hypothesized that 6 weeks of oral Cr supplementation (at a dosage of 1.542 g/kg/day for the first week and 0.385 g/kg/day for following 5 weeks) to female rats would (a) ameliorate the LPS-induced cognitive deficits and (b) increase mTORC1 signaling and markers of synaptic plasticity (pre-synaptic synapsin and post-synaptic PSD-95 proteins) in the dentate gyrus. We also tested the effects of Cr supplementation without LPS to further explore the cognitive effects and mechanisms associated with Cr supplementation.

## 2. Materials and Methods

### 2.1. Animals and Experimental Design

**Figure 1:**
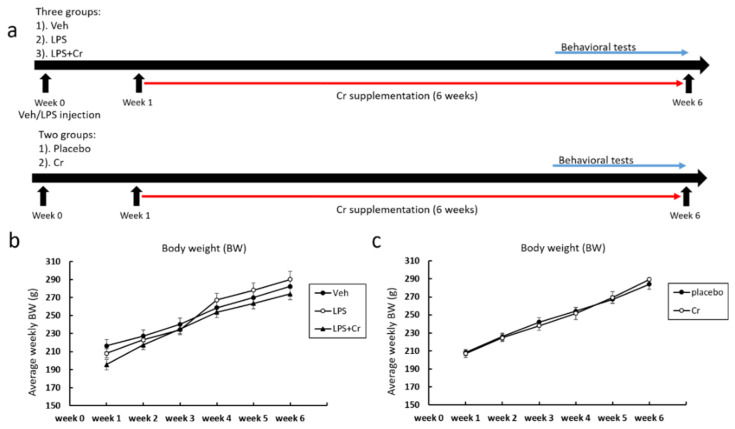
Experimental timeline, changes of bodyweight across the experiment. (**a**) Experimental timeline for the study, where the LPS experiment (experiment 1) has three groups: Veh, LPS and LPS + Cr. All groups underwent either Veh or LPS injection through stereotactic surgery. Creatine (Cr) supplementation or placebo started at week 1 lasting for 6 weeks, and behavioral tests occurred at the last five days of the experiment; Non-LPS (experiment 2) experimental rats were divided into two groups: placebo and Cr group. All rats started Cr or placebo beginning week 1 lasting for 6 weeks, and behavioral tests occurred at the last five days of the experiment. (**b**,**c**) Average weekly BW for each week of the study in both LPS (**b**) and non-LPS (**c**) experiment. Results are presented as mean ± SEM (*n* = 12/group).

Experimental protocols described herein were approved by the University of Missouri Animal Care and Use Committee (protocol code: 10111, date of approval: 11th October 2019). Female Wistar rats (150–200 g, 49 days of age) were bred at the University of Missouri, and individually housed under controlled conditions (12 h: 12 h light/dark cycle, 24 °C). During the entire study, rats were provided food and water (Formulab Diet 5008; Purina, St. Louis, MO, USA) ad libitum. Animals were randomly assigned into specified experimental groups, with each experiment using a separate sample of animals. In experiment 1 (LPS experiment in Figure 1), 7-week-old female rats were randomly divided into three groups to determine whether and how Cr affected cognitive deficits induced by LPS. These groups included: (a) vehicle injected (Veh), (b) LPS injected (LPS), and (c) LPS injected with 6 weeks of oral Cr supplementation (LPS + Cr) (*n* = 12 rats/group). In experiment 2 (non-LPS experiment in Figure 1), 7-week-old female rats were randomly divided into two groups to examine the cognitive and neuro-molecular effects of Cr without LPS. These groups included (a) placebo and (b) oral Cr supplementation (Cr) (*n* = 12 rats/group).

### 2.2. Creatine Supplementation

Creatine (Dymatize, Kings Mountain, NC, USA) was administered to animals daily through drinking water, with standard water utilized as placebo. Notably, fresh bottles were prepared daily. To make the study more similar to human supplementation studies, rats were given a “loading” amount of Cr for the first week at a dosage of 1.542 g/kg per day and, for the following weeks, a maintenance dosage of 0.385 g/kg per day. These doses were based on the normalization of body surface area (BSA) between the rat and human species [22] and were equivalent to an 80 kg human consuming 20 g/day Cr for the first week and 5 g/day Cr for following weeks. Preliminary data were used to determine average daily water consumption for calculating daily Cr administration, and water consumption and body weight were monitored and adjusted throughout the study to confirm proper dosage. 

### 2.3. Surgery and Induction of MCI

The MCI model is discussed in greater detail elsewhere [19,23] and involves intracerebroventricular (i.c.v.) injections of LPS (Sigma, St. Louis, MO, USA). Briefly, rats were anesthetized with 2% isoflurane and positioned into a stereotaxic frame (David Kopf Instruments, Tunjunga, CA, USA). LPS (4.54 μg/μL) or vehicle (sterile saline/10% artificial cerebrospinal fluid) was loaded into a 25 μL Hamilton syringe (Hamilton Co., Renom, NV, USA). To perform the injections, syringes were mounted to an infusion pump (Harvard Apparatus, Holliston, MA, USA), and injectors were positioned into lateral ventricles using the coordinates (in mm relative to Bregma): anteroposterior 0.8, mediolateral ±1.5, and dorsoventral −3.8. Injections performed bilaterally were controlled at a rate of 1 μL/min for a total of 5 min (45.4 μg LPS total per animal) and, following injections, injectors remained in place for another 5 min to ensure that LPS/vehicle was properly diffused. After the successful completion of injections, incisions were closed with tissue adhesive (Vetbond, 3M, Maolewood, MN, USA) and rats were allowed to recover on a 32 °C heating pad until ambulatory. Rats were then returned to their individual home cages for continued monitoring. 

### 2.4. Behavioral Tests

#### 2.4.1. Barnes Maze Test

In order to measure the spatial memory and learning ability of rats, Barnes maze testing was performed. The apparatus for Barnes maze was comprised of a rotating circular gray platform (122 cm in diameter) with 20 holes (each being 10 cm in diameter) evenly distributed. A dark escape box (30 cm in length × 15 cm wide × 13 cm height) was placed beneath one of the 20 holes. The design of the Barnes maze apparatus was based on rodents’ aversion to an open field and to allow rodents to learn and memorize the location of the escape box [24]. In the testing room, temperature, sound, and light were controlled throughout the whole experiment. Testing was performed during the rats’ light cycle for five consecutive days with two trials per day, which is a standard setup of Barnes maze test protocol for rats [24]. Before the first trial on the first day of testing, each rat was gently placed into the escape box for 2 min to associate the escape box as a safe environment. When each trial of Barnes maze began, rats were first placed in the center of the platform covered by a start box for 30 s and then allowed to freely explore the platform for 5 min. Each trial ended when the rat entered the escape box on its own or the 5 min trial ended. If the rat did not find or enter the escape box at the end of the 5 min trial, it was gently guided into the escape box. Once the rat entered escape box, it was allowed to stay inside for another 30 s in order to reinforce the association between escape box and safe environment. After the completion of each trial, rats were returned back to their home cages. The platform was designed in a fashion to eliminate odor cues between rats, and 70% ethanol was also used to deodorize the platform between rats. During the testing phase, the time cost to locate and step into the escape box (latency) and total amount of errors made (nose pokes into holes without the escape box underneath) were recorded. AnyMaze tracking software (Stoelting, Wood Dale, IL, USA) was utilized to record Barnes Maze testing data. 

#### 2.4.2. Novel Object Recognition Test

To evaluate the recognition memory of rats, the novel object recognition test was performed. The novel object recognition test was based on the innate preference of rodents to explore a new object rather than a familiar one, thus allowing for the testing of memory after initial exposure to an object for familiarization [25]. Testing was comprised by three consequent phases: habituation, familiarization, and testing. During the habituation phase, rats were allowed to freely explore the testing field (60 cm in length × 60 cm width × 46 cm height) for 5 min without any object presented. Familiarization occurred 24 h after habituation where rats were given a maximum 10 min to explore two identical objects, which were set 20 cm away from each other and 5-cm away from one wall. Then, testing took place one hour later at which rats were returned back to the testing field with one of familiar objects replaced by a novel object. Again, a maximum 10 min was given during the testing phase. The experiment was stopped when rats had explored objects for a total of 20 s or when the 10 min time period was over. AnyMaze tracking software was used to record the time spent on exploring either object. Temperature, sound, and light were controlled throughout the whole experiment, and upon the completion of each testing, 70% ethanol was used to eliminate the odor. 

### 2.5. Euthanasia and Tissue Harvesting

Experimental rats were euthanized on next following day of the last day of the behavioral test via carbon dioxide asphyxiation, followed by quick removal of their brains. Dentate gyrus punches that were 2 mm thick and 3 mm in diameter were taken from coronal brain slices by using a brain matrix (Braintree, Braintree, MA, USA). Isolated dentate gyrus punches were then frozen with liquid nitrogen and stored at −80 °C until processing.

### 2.6. RNA Isolation, cDNA Synthesis, and Real-Time Polymerase Chain Reaction (RT-PCR)

RNA isolation, cDNA synthesis, and RT-PCR were performed as described before by our laboratory [26]. Briefly, dentate gyrus punches were placed in TRIzol (Invitrogen, Carlsbad, CA, USA) with RNase-free stainless beads and homogenized for 1 min at 25 Hz for three times via the Tissuelyer (Qiagen, Germantown, MD, USA). The TRIzol protocol was then carried out according to manufacturer’s instructions to obtain an RNA pellet. RNA pellets were dissolved in RNase-free water for quantification by using the Nanodrop 1000 (Thermo Scientific, Waltham, MA, USA). Prior to cDNA synthesis, RNA was treated with DNase I (Thermo Scientific, Waltham, MA, USA) followed by DNase I inactivation with EDTA for 10 min at 65 °C. Thereafter, DNA-free RNA was reverse transcribed using a High Capacity cDNA Reverse Transcription Kit (Applied Biosystems, Carlsbad, CA, USA). For RT-PCR, 15 μg of cDNA from each sample was assayed in duplicate by using gene-specific primers (Table 1) and SYBR green Supermix (Bio-Rad Laboratories, Hercules, CA, USA). mRNA expression values were presented as 2^ΔCT^ whereby ΔCT = 18S CT-gene of interest CT. 

### 2.7. Immunoblotting

Immunoblotting was performed as previously described [26] to examine select cell signaling and synaptic protein markers in the dentate gyrus after the experimental interventions described above. In short, dentate gyrus tissue was homogenized in Radioimmunoprecipitation (RIPA) buffer [50 mm Tris-HCl (pH 8.0), 150 mm NaCl, 1% NP-40, 0.5% sodium deoxycholate, 1% SDS, 1 × protease + phosphatase inhibitor cocktail] using a Tissuelyser (Qiagen) for 1 min at 25 Hz for three times. The homogenate was then centrifuged at 12,000× *g* for 10 min, and the supernatant was extracted. Protein concentrations were then determined through the bicinchoninic acid (BCA) assay (Piece Biotechnology, Rockford, IL, USA). Then, 27 μg of protein from each sample was loaded onto 4–15% Criterion TGX gels (Bio-Rad, Hercules, CA, USA), and electrophoresis was ran at 200 V for 1 h. Proteins were then transferred onto polyvinylidene fluoride (PVDF) membranes (Bio-Rad, Hercules, CA, USA) and incubated with Ponceau S (Sigma, St Louis, MO, USA) to verify the equal loading among all lanes. After that, 5% nonfat milk in Tris-buffered saline + 0.1%, Tween20 (TBS-T) was used as a blocking agent. Primary antibodies (rabbit polyclonal) for glial fibrillary acidic protein (GFAP) (dilution 1:2000; catalog #12389, Cell Signaling, Danvers, MA, USA), phosphorylated 70kDa ribosomal protein S6 kinase (p-p70S6K) (dilution 1:1000, catalog #9205, Cell Signaling, Danvers, MA, USA), 70kDa ribosomal protein S6 kinase (p70S6K) (dilution 1:1000, catalog #9202, Cell Signaling, Danvers, MA, USA)), phosphorylated mammalian target of rapamycin (p-mTOR) (dilution 1:1000, catalog #2971, Cell Signaling, Danvers, MA, USA), mammalian target of rapamycin (mTOR) (dilution 1:1000, catalog #2972, Cell Signaling, Danvers, MA, USA), postsynaptic density protein-95 (PSD-95) (dilution 1:1000, catalog #3409, Cell Signaling, Danvers, MA, USA), Synapsin (dilution 1:1000, catalog #2312, Cell Signaling, Danvers, MA, USA), glyceraldehyde 3-phosphate dehydrogenase (GAPDH) (dilution 1:20,000, catalog #5174, Cell Signaling, Danvers, MA, USA) were diluted in TBS-T with 5% BSA and applied to membranes overnight at 4 °C. The following day, horseradish peroxidase (HRP)-conjugated secondary antibodies (dilution 1:1000; Cell Signaling) diluted in TBS-T with 5% non-fat milk were applied to membranes for one hour at room temperature. Next, enhanced chemiluminescence (ECL) substrate solution (Pierce Biotechnology, Biotechnology, Rockford, IL, USA) was applied to membranes for two minutes. A gel documentation system (Kodak 4000R imager and Molecular Imagery Software; Kodak Molecular Imaging Systems, New Haven, CT, USA) was then used to capture digital images of each membrane. Associated software was used to determine band densities, and these values were normalized to the values of GAPDH to obtain final expression values. 

### 2.8. Statistical Analysis

All analytical procedures were performed using SigmaPlot, version 14.0 (Systat Software, Inc., Chicago, IL, USA). Two-way ANOVAs [Trials (Trial1-10) × Treatment (Veh vs. LPS vs. LPS + Cr)] were performed on dependent variables obtained from the LPS experiment. Likewise, two-way ANOVAs [Trials (Trial1-10) × Treatment (Placebo vs. Cr)] were performed on dependent variables from the non-LPS experiment. Mauchly’s tests of sphericity were performed on latency time and errors, and both tests yield *p* value < 0.05. Thus, Greenhouse–Geisser corrections were applied when reporting the trial and interaction *p* values. Holm–Šídák post hoc analyses were applied for multiple comparisons. Statistical analyses on mRNA expression, band densities, and novel object recognition test were conducted using one-way ANOVAs followed by Holm–Šídák post hoc analyses for the LPS experiment. Student’s *t* tests were used to assess group differences for the non-LPS experiment. All values are expressed as the mean ± SEM, and statistical significance was established as *p* ≤ 0.05 for all analyses.

## 3. Results

### 3.1. Experiment 1

**Figure 2:**
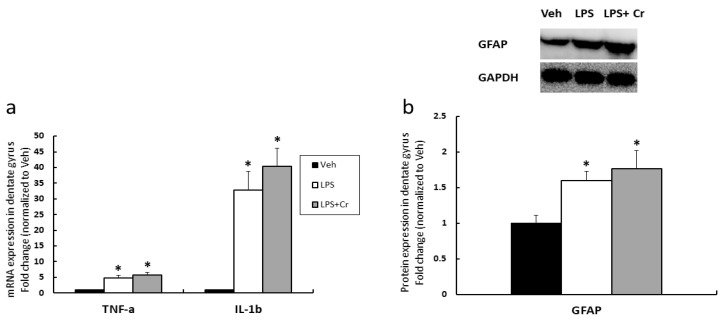
Proinflammatory markers and marker for the activation of astrocytes in the dentate gyrus of Veh, LPS, and LPS + Cr groups. (**a**) Proinflammatory TNF-α, IL-1β mRNA expression level in the dentate gyrus. (**b**) GFAP protein expression in the dentate gyrus. Results are presented as mean ± SEM (*n* = 8). * *p* < 0.05 significantly different from Veh group.

In order to verify LPS-induced inflammation in the dentate gyrus, we performed RT-PCR to examine pro-inflammatory genes and western blot for GFAP protein expression (Figure 2). For TNF-α mRNA (Figure 2a), Veh vs. LPS was 1.00 ± 0.08 vs. 4.85 ± 0.83-fold, respectively, and Veh vs. LPS + Cr was 1.00 ± 0.08 vs. 5.75 ± 0.86-fold, respectively (*p* < 0.0001 for both comparisons). For IL-1β mRNA (Figure 2a), Veh vs. LPS was 1.00 ± 0.09 vs. 32.81 ± 6.01-fold, respectively, and Veh vs. LPS + Cr was 1.00 ± 0.09 vs. 40.33 ± 5.75-fold, respectively (*p* < 0.0001 for both comparisons). For GFAP protein (Figure 2b), Veh vs. LPS was 1.00 ± 0.11 vs. 1.59 ± 0.13-fold, respectively (*p* = 0.004), and Veh vs. LPS + Cr was 1.00 ± 0.11 vs. 1.76 ± 0.25-fold, respectively (*p* = 0.006). There was no significant difference observed between LPS and LPS + Cr group for the assayed pro-inflammatory mRNA markers (*p* = 0.244 for TNF-α mRNA, *p* = 0.408 for IL-1β mRNA) or GFAP protein levels (*p* = 0.743). Overall, these data suggest that LPS injection was sufficient to induce neuro-inflammation in the dentate gyrus.

**Figure 3:**
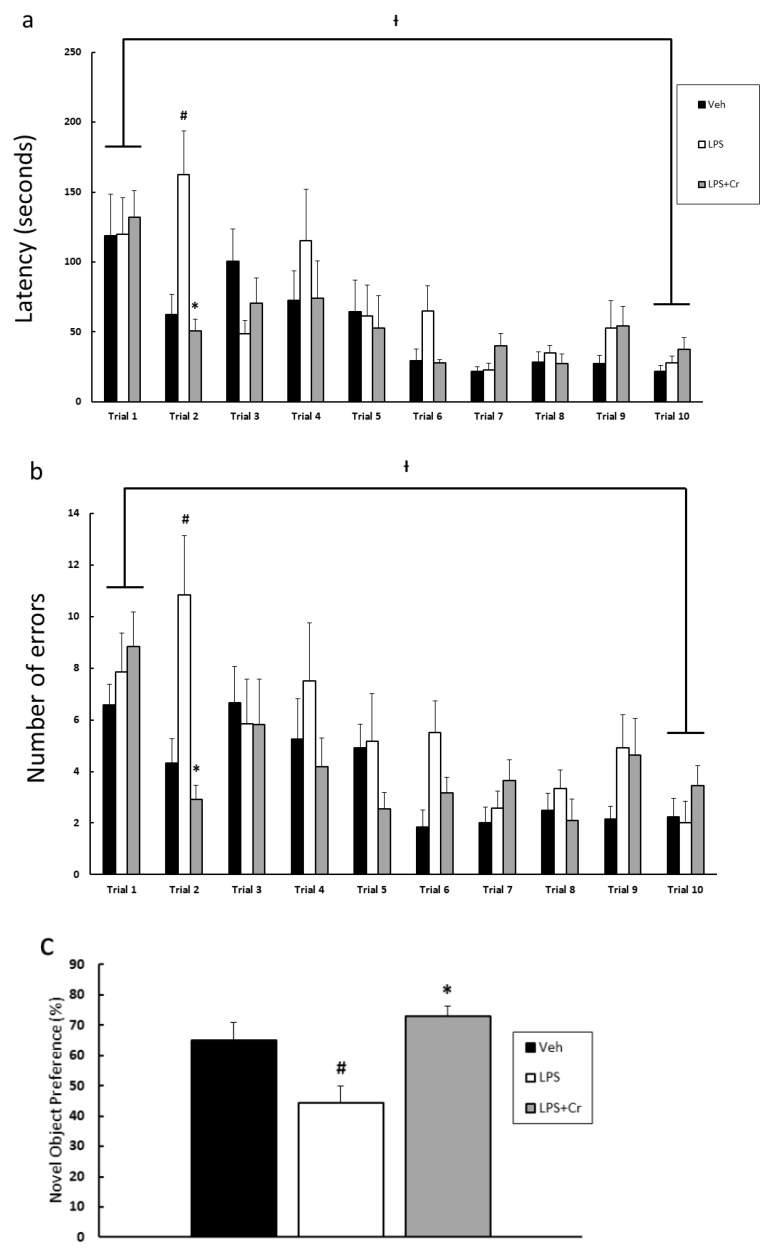
Effects of 6 weeks of creatine (Cr) on spatial memory and object recognition memory deficits induced by LPS. (**a**) Average latency time (seconds) to locate the escape box during five days of Barnes maze testing (2 trials/day, 10 trials in total). (**b**) Number of errors made before locating the escape box during five days of Barnes maze testing (2 trials/day, 10 trials in total). (**c**) Preference of novel object (%) during the Novelty object recognition test. Results are presented as mean ± SEM (*n* = 12). ⟊ *p* < 0.05 significantly different from trial 1 for both Veh, LPS, and LPS + Cr groups. # *p* < 0.05 significantly different from Veh group. * *p* < 0.05 significantly different from LPS group.

The effects of Cr supplementation on spatial memory deficit induced by LPS is presented in Figure 3a,b. Significant interactions existed between group and trials for both latency (F (9152) = 2.105, *p* = 0.030) and errors (F (10,170) = 1.861, *p* = 0.049) during the Barnes Maze testing. During trial 2, which followed the initial trial (trial 1), the LPS group revealed a higher average latency time (in seconds) of locating the escape box (Figure 3a), compared with both Veh and LPS + Cr group; Veh vs. LPS was 62.5 ± 14.3 vs. 162.4 ± 31.2, respectively (*p* = 0.0001); LPS vs. LPS + Cr was 162.4 ± 31.2 vs. 50.7 ± 8.5, respectively (*p* < 0.0001). No significant difference between Veh and LPS + Cr (*p* = 0.644) suggests that LPS induced a spatial memory deficit shown by a slower rate of learning Barnes maze test in contrast to the Veh, and this deficit was attenuated with Cr supplementation. The LPS group also exhibited a higher number of errors made before locating the escape box at the trial 2, compared with both Veh and LPS + Cr group (Figure 3b), Veh vs. LPS was 4.3 ± 0.9 vs. 10.8 ± 2.3, (*p* = 0.0003), and LPS vs. LPS + Cr was 10.8 ± 2.3 vs. 2.9 ± 0.6, (*p* < 0.0001). Similar to latency, there was not a significant effect between Veh and LPS + Cr group (*p* = 0.412). Interestingly, all groups significantly decreased their latency and number of errors between trial 1 to trial 10 (*p* < 0.0001, Figure 3a,b), indicating intact spatial memory learning in all groups. Taken together, these data suggest that Cr was able to ameliorate the spatial memory deficit induced by LPS by undergoing the spatial learning at a faster rate. Data in Figure 3c show results from the novel object recognition test. In contrast to the Veh and LPS + Cr group, the LPS group showed a significantly decreased preference (in percent) to the novel object (Figure 3c), Veh vs. LPS was 0.65 ± 0.06 vs. 0.44 ± 0.05 (*p* = 0.015), and LPS vs. LPS + Cr was 0.44 ± 0.05 vs. 0.73 ± 0.04, respectively (*p* = 0.002). There was no significant effect between Veh and LPS + Cr detected (*p* = 0.260). Again, these data suggest that Cr supplementation was able to ameliorate the recognition deficit induced by LPS.

**Figure 4:**
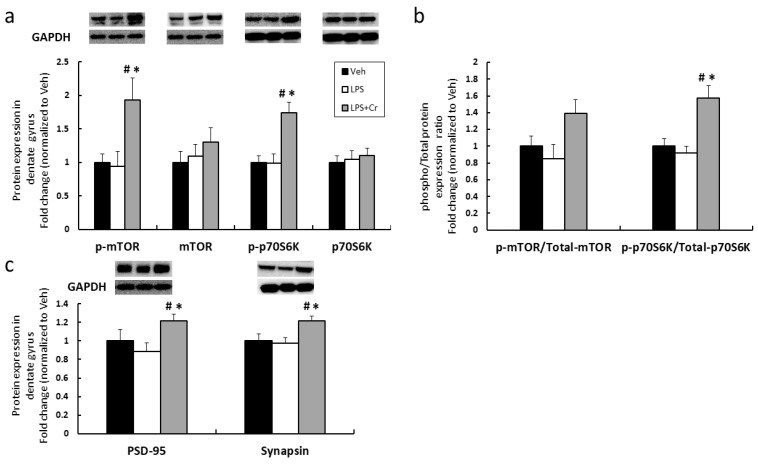
Effects of 6 weeks of Cr supplementation on molecular signaling in dentate gyrus following LPS. (**a**) Proteins expression level of mTORC1 signaling pathway. (**b**) Ratio of phosphorylation/total mTORC1 signaling proteins. (**c**) Synaptic proteins downstream to the mTORC1 signaling. Results are presented as mean ± SEM (*n* = 8). # *p* < 0.05 significantly different from Veh group. * *p* < 0.05 significantly different from LPS group.

To detect the molecular signaling changes in the dentate gyrus induced by 6 weeks of Cr supplementation, we next examined the dentate gyrus for mTORC1 signaling markers as well as select synaptic proteins (Figure 4). Both the Veh and LPS groups presented the similar expression patterns of all assayed mTORC1 signaling proteins. However, when adding Cr to LPS, LPS + Cr group had significantly higher expression patterns versus the Veh and LPS groups. Pathway increases were (a) 1.93 ± 0.33-fold to Veh for mTOR protein phosphorylation (*p* = 0.025 to Veh, *p* = 0.026 to LPS); (b) 1.73 ± 0.16-fold to Veh for p70S6K protein phosphorylation (*p* = 0.003 to Veh, *p* = 0.002 to LPS); (c). 1.22 ± 0.07-fold to Veh for PSD-95 protein (*p* = 0.033 to Veh, *p* = 0.015 to LPS); and (d) 1.22 ± 0.05-fold to Veh for synapsin protein (*p* = 0.026 to Veh, *p* = 0.008 to LPS) (Figure 4). Of note, there was no significant difference between groups for total proteins (Figure 4a). Taken together, these data suggest that 6 weeks of Cr supplementation increased mTORC1 signaling and the expression of select synaptic proteins in the dentate gyrus.

### 3.2. Experiment 2

**Figure 5:**
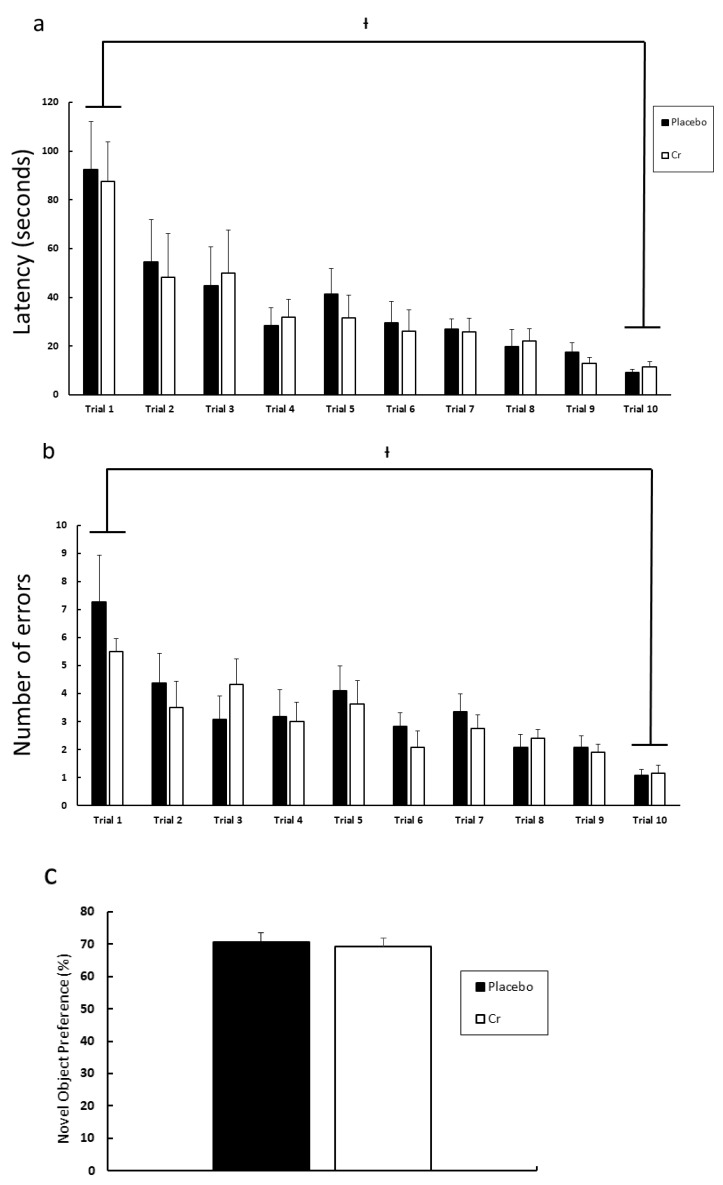
Effects of 6 weeks of Cr supplementation on spatial memory and recognition memory without LPS. (**a**) Average latency time (seconds) to locate the escape box during five days of Barnes maze testing (2 trials/day, 10 trials in total). (**b**) Number of errors made before locating the escape box during five days of Barnes maze testing (2 trials/day, 10 trials in total). (**c**) Preference of novel object (%) during the Novel object recognition test. Results are presented as mean ± SEM (*n* = 12). ⟊ *p* < 0.05 significantly different from trial 1 for both placebo and Cr groups.

Experiment 2 was performed without the surgical injection of LPS in order to determine if Cr supplementation alone affected some of the aforementioned performance outcomes and molecular variables. The Barnes maze and novel object recognition testing were performed for both placebo (control) and Cr supplementation (Cr) groups (Figure 5). For the Barnes maze testing, two-way ANOVA revealed that there was not a statistically significant interaction between groups and trials for both latency and errors (Figure 5a,b). However, a statistically significant difference existed between trial 1 and trial 10 within the placebo and Cr groups, with *p* < 0.0001 for both latency and errors (Figure 5a,b). For the novel object recognition test, there was no significant group difference (*p* = 0.740) observed (Figure 5c), suggesting that Cr supplementation did not affect test outcomes. Taken altogether, these data suggest that 6 weeks of Cr supplementation was not able to further enhance cognition under the condition without LPS.

**Figure 6:**
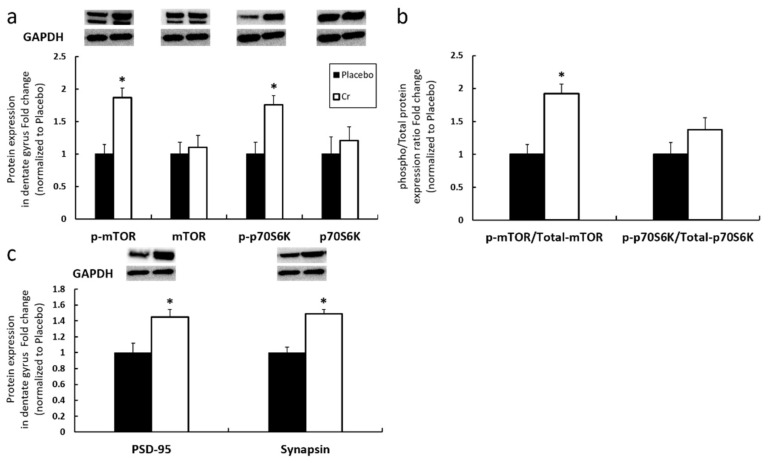
Effects of 6 weeks of Cr supplementation on molecular signaling in dentate gyrus without LPS. (**a**) Proteins expression level of mTORC1 signaling pathway targets. (**b**) Ratio of phosphorylation/total mTORC1 signaling proteins. (**c**) synaptic proteins downstream of mTORC1 signaling. Results are presented as mean ± SEM (*n* = 8). * *p* < 0.05 significantly different from placebo group.

Western blotting was performed to examine the protein expression mTORC1 signaling markers and downstream synaptic proteins in the dentate gyrus between the placebo and Cr groups (Figure 6). In contrast to the placebo group, Cr group revealed significantly increased mTOR phosphorylation (placebo vs. Cr was 1.00 ± 0.15 vs. 1.87 ± 0.15-fold, respectively, *p* = 0.003), p70S6K phosphorylation (placebo vs. Cr was 1.00 ± 0.18 vs. 1.76 ± 0.14-fold, respectively, *p* = 0.006) (Figure 6a), PSD-95 (placebo vs. Cr was 1.00 ± 0.13 vs. 1.45 ± 0.18-fold, respectively, *p* = 0.039), synapsin (placebo vs. Cr was 1.00 ± 0.23 vs. 1.49 ± 0.10-fold, respectively, *p* = 0.030) (Figure 6c). There were no significant changes observed for total proteins (*p* = 0.692 for mTOR, *p* = 0.553 for p70S6K) (Figure 6a). Additionally, the ratio between p-mTOR/mTOR was significantly higher in Cr group in contrast to placebo group (placebo vs. Cr was 1.00 ± 0.26 vs. 1.92 ± 0.21-fold, respectively, *p* = 0.008) (Figure 6b). Again, these data suggest that Cr was able to elevate the mTORC1 signaling and the expression of synaptic proteins.

## 4. Discussion

Several studies have suggested that creatine (Cr) is a candidate for enhancing cognition [7,8]. Herein, we tested the hypothesis that oral administration of Cr would ameliorate cognitive deficits induced by LPS in female rats. Our data suggest that 6 weeks of Cr supplementation ameliorated spatial and recognition memory deficits in the presence of LPS. Additionally, we found that ameliorated cognition was concurrent with an increase in mTORC1 signaling within the dentate gyrus. We have also identified that spatial and recognition memory were not further enhanced by Cr supplementation in the absence of LPS, even though mTORC1 signaling was still elevated within the dentate gyrus. 

In experiment 1, we utilized LPS to mimic mild cognitive impairment (MCI), given that this model is known to cause neuro-inflammation and cognitive dysfunction [27,28,29]. To verify the presence of neuro-inflammation within the dentate gyrus of LPS injected rats, proinflammatory transcripts were quantified through RT-PCR. Similar to other findings [28], our results confirmed that TNF-α and IL-1β mRNAs in dentate gyrus were significantly increased by i.c.v. LPS injections relative to the Veh group. However, Cr supplementation failed to decrease the increased expression of inflammatory transcripts induced by LPS. Notably, this finding does not agree with prior evidence suggesting Cr supplementation has anti-inflammatory effects [30]. The mechanism by which LPS induces pro-inflammatory cytokine production in the brain is through NF-κB signaling activation, caused by toll-like-receptor 4 (TLR-4) activation in microglia [28]. However, we did not perform experiments to examine this signaling in microglia. Thus, this needs to be further investigated. We also assayed glial fibrillary acidic protein (GFAP) levels as a marker for reactive astrocytes [31]. Consistent to our proinflammatory mRNA data, GFAP protein was elevated in all LPS injected groups, regardless of the Cr supplementation. Proinflammatory cytokine (e.g., TNF-α) secretion following LPS are known to trigger reactive astrocytes [32]. Based on our data, it is possible to speculate that activated astrocytes lead to amplified neuro-inflammation and cause functional changes within the neuro-environment [33]. However, again, Cr did not seem to mitigate this effect. Overall, our data suggest that LPS was sufficient to induce the neuro-inflammation for studying cognitive impairment in the current study; however, Cr seemingly ameliorates cognitive impairment through non-inflammatory pathways. 

The Barnes maze and novel object recognition tests were performed to evaluate the cognitive effects of Cr in the presence of LPS, as they strongly correlate with hippocampus-dependent memory [24,25]. According to our behavioral data, Cr supplementation ameliorated the impaired acquisition of spatial learning task compared to the LPS only group, shown by its shortened latency and less errors made to reach the escape box at trial 2 of the training regime. Additionally, all groups of rats in the Barnes maze test, including the LPS group, improved their spatial memory from the first trial of their training, implying that the enhanced spatial memory caused by Cr could have resulted from an improved learning ability in contrast to the LPS group. In the object recognition test, Cr supplementation ameliorated recognition memory compared to the LPS only group shown by the increased amount of time taken to explore the new object, which provides a reliable index of recognition memory [25]. This finding agrees with both animal and human studies regarding the ability of Cr supplementation to affect cognition [10,34,35]. However, evidence regarding whether Cr is sufficient to ameliorate cognitive impairment in disease models are relatively lacking. Therefore, our study provides preliminary data in this area and warrants future research. 

Our finding of upregulated mTORC1 signaling concurrent with ameliorated cognition caused by Cr supplementation is in line with the notion that mTORC1 signaling plays an essential role in regulating learning and memory [36]. Numerous behavioral studies have reported that the selective inhibition of mTORC1 by rapamycin impairs learning and memory formation [37,38,39]. Mechanistically, studies indicate the involvement of mTORC1 signaling in regulating long-term potentiation in the hippocampus [15,40], an event that links modifications in synaptic strength to long-lasting behavioral changes. Thus, the regulation of synaptic plasticity via mTORC1 signaling may underlie hippocampus-dependent learning and memory formation. The synapse-associated proteins, particularly the pre-synaptic synapsin and post-synaptic PSD-95, promote synaptic plasticity and neuronal excitability [41,42]. In line with our finding of increased mTORC1 signaling, we also found upregulation of synapsin and PSD-95 proteins expression with Cr supplementation. Overall, our above findings suggest that mTORC1 signaling may be critical for Cr to exert its effects to overcome the LPS-induced cognitive impairment by promoting synaptic function and plasticity. The upstream mechanism that facilitates activation of mTORC1 via Cr supplementation was not investigated in this study. However, it is possible that Cr upregulates mTORC1 signaling by stimulating insulin-like growth factor (IGF-1) secretion, which binds to the IGF-1 receptor and triggers downstream phosphatidylinositol-3-kinase (PI3K)-AKT signaling activation leading to the phosphorylation of mTOR [43,44]. Noticeably, there is a conflicting role regarding the mTORC1 signaling in mediating cognition. In the aging brain, pathological hyperactivation of mTORC1 leads to the accumulation of β-amyloid peptide (Aβ) and dysregulated autophagy, fostering the cognitive impairment that is associated with neurodegenerative diseases [45]. Therefore, precise regulation of mTORC1 signaling may be required for different stages of life or diseases. Taken together, ameliorated cognitive impairment via Cr supplementation with a concomitant upregulation of mTORC1 signaling within the dentate gyrus suggests a potential link. Further research will be required to investigate whether mTORC1 signaling is required for Cr to fully exert its cognitive effects.

Experiment 2 examined whether Cr enhances spatial memory and recognition memory in the absence of LPS. Our data suggest that Cr supplementation for 6 weeks failed to enhance both spatial and recognition memory compared to placebo group. This result conflicts with other findings in which 6 weeks of Cr supplementation was reported to enhance spatial learning and memory in 7-months-old wild-type (WT) mice as assessed with the Morris water maze (MWM) test [46]. Additionally, our findings do not agree with data suggesting Cr supplementation enhances recognition memory in healthy aged mice [34]. However, tests performed by those studies were carried out at an older stage of life compared to the current study. It is possible that those conflicting outcomes are a result of different age of animals being utilized. In our study, animals supplemented with Cr without LPS injections may have a “ceiling effect” for cognition due to their young age that prevent them from being further affected with Cr supplementation. As supported by other studies, Cr may only improve cognition when cognitive function is impaired [47]. Interestingly, in the current study, Cr supplementation still augments mTORC1 signaling within the dentate gyrus, even though the cognitive function was not altered.

A limitation of the present study is that the estrus cycle was not controlled throughout the experiment, and this has been shown to influence cognition [48]. Future studies are also needed to investigate whether mTORC1 signaling is required for Cr to fully exert its effects in the presence of LPS. Additionally, an optimal Cr dosing protocol for improving cognition needs to be studied given that heterogenous dosages exist in the literature. A key limitation to our cognitive tests is that, although Barnes maze and novel object recognition tests were performed to evaluate spatial and recognition memory, there are still other areas of cognitive functioning remained to be tested in order to gain a more comprehensive understanding of the cognitive effects of Cr supplementation.

## 5. Conclusions

In summary, we show that 6 weeks of Cr supplementation ameliorates cognitive deficits induced by LPS in female rats, potentially through the activation of mTORC1 signaling and the upregulation of synaptic proteins within the dentate gyrus. While 6 weeks of Cr supplementation does not further enhance cognitive function in the absence of LPS, there is a concurrent upregulation of mTORC1 signaling and synaptic proteins within the dentate gyrus. As a result, our preclinical findings may pave the way for future investigations of neurocognitive effects of Cr in MCI patients. 

## Figures and Tables

**Table 1:** Primers used for RT-PCR.

Gene	Forward (5′→3′)	Reverse (5′→3′)	Accession No.
18S	GCTCGCTCCTCTCCTACTTG	GATAAATGCACGCGTTCCCC	NR_046237.2
TNF-α	AACACACGAGACGCTGAAGT	TCCAGTGAGTTCCGAAAGCC	NM_012675.3
IL-1β	TGACTTCACCATGGAACCCG	GACCTGACTTGGCAGAGGAC	NM_031512.2

## Data Availability

The data that support the findings of this study are available on request from the corresponding author (F.W.B.).

## References

[1] Alzheimer’s Association (2021). 2021 Alzheimer’s disease facts and figures. Alzheimers Dement..

[2] Collie A., Maruff P. (2000). The neuropsychology of preclinical Alzheimer’s disease and mild cognitive impairment. Neurosci. Biobehav. Rev..

[3] Xu H., Yang R., Dintica C.S., Qi X., Song R., Bennett D.A., Xu W. (2020). Association of lifespan cognitive reserve indicator with the risk of mild cognitive impairment and its progression to dementia. Alzheimers Dement..

[4] Long J.M., Holtzman D.M. (2019). Alzheimer Disease: An Update on Pathobiology and Treatment Strategies. Cell.

[5] Serrano-Pozo A., Frosch M.P., Masliah E., Hyman B.T. (2011). Neuropathological Alterations in Alzheimer Disease. Cold Spring Harb. Perspect. Med..

[6] Mujika I., Padilla S. (1997). Creatine Supplementation as an Ergogenic Aid for Sports Performance in Highly Trained Athletes: A Critical Review. Int. J. Sports Med..

[7] Avgerinos K.I., Spyrou N., Bougioukas K.I., Kapogiannis D. (2018). Effects of creatine supplementation on cognitive function of healthy individuals: A systematic review of randomized controlled trials. Exp. Gerontol..

[8] Owen L., Sunram-Lea S.I. (2011). Metabolic Agents that Enhance ATP can Improve Cognitive Functioning: A Review of the Evidence for Glucose, Oxygen, Pyruvate, Creatine, and l-Carnitine. Nutrients.

[9] Rae C., Digney A.L., McEwan S.R., Bates T.C. (2003). Oral creatine monohydrate supplementation improves brain performance: A double-blind, placebo-controlled, cross-over trial. Proc. R. Soc. B Boil. Sci..

[10] McMorris T., Mielcarz G., Harris R.C., Swain J.P., Howard A.N. (2007). Creatine Supplementation and Cognitive Performance in Elderly Individuals. Aging Neuropsychol. Cogn..

[11] McMorris T., Harris R.C., Swain J., Corbett J., Collard K., Dyson R.J., Dye L., Hodgson C., Draper N. (2006). Effect of creatine supplementation and sleep deprivation, with mild exercise, on cognitive and psychomotor performance, mood state, and plasma concentrations of catecholamines and cortisol. Psychopharmacology.

[12] McMorris T., Harris R., Howard A., Langridge G., Hall B., Corbett J., Dicks M., Hodgson C. (2007). Creatine supplementation, sleep deprivation, cortisol, melatonin and behavior. Physiol. Behav..

[13] Turner C., Byblow W., Gant N. (2015). Creatine Supplementation Enhances Corticomotor Excitability and Cognitive Performance during Oxygen Deprivation. J. Neurosci..

[14] Laplante M., Sabatini D.M. (2009). mTOR signaling at a glance. J. Cell Sci..

[15] Tang S.J., Reis G., Kang H., Gingras A.-C., Sonenberg N., Schuman E.M. (2002). A rapamycin-sensitive signaling pathway contributes to long-term synaptic plasticity in the hippocampus. Proc. Natl. Acad. Sci. USA.

[16] Bekinschtein P., Katche C., Slipczuk L.N., Igaz L.M., Cammarota M., Izquierdo I., Medina J.H. (2007). mTOR signaling in the hippocampus is necessary for memory formation. Neurobiol. Learn. Mem..

[17] Pazini F.L., Cunha M., Rosa J., Colla A.R.S., Lieberknecht V., Oliveira-Giacomelli Á., Rodrigues A.L.S. (2016). Creatine, similar to Ketamine, Counteracts Depressive-Like Behavior Induced by Corticosterone via PI3K/Akt/mTOR Pathway. Mol. Neurobiol..

[18] Pazini F.L., Rosa J.M., Camargo A., Fraga D.B., Moretti M., Siteneski A., Rodrigues A.L.S. (2020). mTORC1-dependent signaling pathway underlies the rapid effect of creatine and ketamine in the novelty-suppressed feeding test. Chem. Interact..

[19] Kelty T.J., Schachtman T.R., Mao X., Grigsby K.B., Childs T.E., Olver T.D., Michener P.N., Richardson R.A., Roberts C.K., Booth F.W. (2019). Resistance-exercise training ameliorates LPS-induced cognitive impairment concurrent with molecular signaling changes in the rat dentate gyrus. J. Appl. Physiol..

[20] Jessberger S., Clark R.E., Broadbent N.J., Clemenson J.G.D., Consiglio A., Lie D.C., Squire L.R., Gage F.H. (2009). Dentate gyrus-specific knockdown of adult neurogenesis impairs spatial and object recognition memory in adult rats. Learn. Mem..

[21] Hainmueller T., Bartos M. (2020). Dentate gyrus circuits for encoding, retrieval and discrimination of episodic memories. Nat. Rev. Neurosci..

[22] Reagan-Shaw S., Nihal M., Ahmad N. (2008). Dose translation from animal to human studies revisited. FASEB J..

[23] Grigsby K.B., Ruegsegger G.N., Childs T.E., Booth F.W. (2018). Overexpression of Protein Kinase Inhibitor Alpha Reverses Rat Low Voluntary Running Behavior. Mol. Neurobiol..

[24] Gawel K., Gibula E., Marszalek-Grabska M., Filarowska J., Kotlinska J.H. (2019). Assessment of spatial learning and memory in the Barnes maze task in rodents—methodological consideration. Naunyn-Schmiedebergs Arch. Pharmacol..

[25] Leger M., Quiedeville A., Bouet V., Haelewyn B., Boulouard M., Schumann-Bard P., Freret T. (2013). Object recognition test in mice. Nat. Protoc..

[26] Ruegsegger G.N., Toedebusch R.G., Childs T.E., Grigsby K.B., Booth F.W. (2016). Loss of Cdk5 function in the nucleus accumbens decreases wheel running and may mediate age-related declines in voluntary physical activity. J. Physiol..

[27] Lee J.W., Lee Y.K., Yuk D.Y., Choi D.Y., Ban S.B., Oh K.W., Hong J.T. (2008). Neuro-inflammation induced by lipopolysaccharide causes cognitive impairment through enhancement of beta-amyloid generation. J. Neuroinflamm..

[28] Zhao J., Bi W., Xiao S., Lan X., Cheng X., Zhang J., Lu D., Wei W., Wang Y., Li H. (2019). Neuroinflammation induced by lipopolysaccharide causes cognitive impairment in mice. Sci. Rep..

[29] Perez-Dominguez M., Ávila-Muñoz E., Domínguez-Rivas E., Zepeda A. (2019). The detrimental effects of lipopolysaccharide-induced neuroinflammation on adult hippocampal neurogenesis depend on the duration of the pro-inflammatory response. Neural Regen. Res..

[30] Dean P.J.A., Arikan G., Opitz B., Sterr A. (2017). Potential for use of creatine supplementation following mild traumatic brain injury. Concussion.

[31] Zhang S., Wu M., Peng C., Zhao G., Guanjie Z. (2017). GFAP expression in injured astrocytes in rats. Exp. Ther. Med..

[32] Liddelow S.A., Guttenplan K.A., Clarke L.E., Bennett F.C., Bohlen C.J., Schirmer L., Bennett M.L., Münch A.E., Chung W.-S., Peterson T.C. (2017). Neurotoxic reactive astrocytes are induced by activated microglia. Nature.

[33] Liu L.-R., Liu J.-C., Bao J.-S., Bai Q.-Q., Wang G. (2020). Interaction of Microglia and Astrocytes in the Neurovascular Unit. Front. Immunol..

[34] Bender A., Beckers J., Schneider I., Hölter-Koch S., Haack T., Ruthsatz T., Vogt-Weisenhorn D., Becker L., Genius J., Rujescu D. (2008). Creatine improves health and survival of mice. Neurobiol. Aging.

[35] Allahyar R., Akbar A., Iqbal F. (2016). Effect of creatine monohydrate supplementation on learning, memory and neuromuscular coordination in female albino mice. Acta Neuropsychiatr..

[36] Graber T.E., McCamphill P., Sossin W.S. (2013). A recollection of mTOR signaling in learning and memory. Learn. Mem..

[37] Parsons R., Gafford G.M., Helmstetter F. (2006). Translational Control via the Mammalian Target of Rapamycin Pathway Is Critical for the Formation and Stability of Long-Term Fear Memory in Amygdala Neurons. J. Neurosci..

[38] Deli A., Schipany K., Rosner M., Höger H., Pollak A., Li L., Hengstschläger M., Lubec G. (2012). Blocking mTORC1 activity by rapamycin leads to impairment of spatial memory retrieval but not acquisition in C57BL/6J mice. Behav. Brain Res..

[39] Jobim P.F., Pedroso T.R., Christoff R.R., Werenicz A., Maurmann N., Reolon G.K., Roesler R. (2012). Inhibition of mTOR by rapamycin in the amygdala or hippocampus impairs formation and reconsolidation of inhibitory avoidance memory. Neurobiol. Learn. Mem..

[40] Ma T., Hoeffer C.A., Capetillo-Zarate E., Yu F., Wong H., Lin M.T., Tampellini D., Klann E., Blitzer R.D., Gouras G.K. (2010). Dysregulation of the mTOR Pathway Mediates Impairment of Synaptic Plasticity in a Mouse Model of Alzheimer’s Disease. PLoS ONE.

[41] Cesca F., Baldelli P., Valtorta F., Benfenati F. (2010). The synapsins: Key actors of synapse function and plasticity. Prog. Neurobiol..

[42] Keith D.J. (2008). Excitation control: Balancing PSD-95 function at the synapse. Front. Mol. Neurosci..

[43] Bond P. (2016). Regulation of mTORC1 by growth factors, energy status, amino acids and mechanical stimuli at a glance. J. Int. Soc. Sports Nutr..

[44] Wrigley S., Arafa D., Tropea D. (2017). Insulin-Like Growth Factor 1: At the Crossroads of Brain Development and Aging. Front. Cell. Neurosci..

[45] Hodges S.L., Reynolds C.D., Smith G.D., Jefferson T.S., Nolan S., Lugo J.N. (2018). Molecular interplay between hyperactive mammalian target of rapamycin signaling and Alzheimer’s disease neuropathology in the NS-Pten knockout mouse model. NeuroReport.

[46] Snow W.M., Cadonic C., Cortes-Perez C., Chowdhury S.K.R., Djordjevic J., Thomson E., Bernstein M.J., Suh M., Fernyhough P., Albensi B.C. (2018). Chronic dietary creatine enhances hippocampal-dependent spatial memory, bioenergetics, and levels of plasticity-related proteins associated with NF-κB. Learn. Mem..

[47] Rawson E.S., Lieberman H.R., Walsh T.M., Zuber S.M., Harhart J.M., Matthews T.C. (2008). Creatine supplementation does not improve cognitive function in young adults. Physiol. Behav..

[48] Woolley C., Gould E., Frankfurt M., McEwen B. (1990). Naturally occurring fluctuation in dendritic spine density on adult hippocampal pyramidal neurons. J. Neurosci..

